# Supplementary material for: Unconventional polarization fatigue in van der Waals layered ferroelectric ionic conductor CuInP2S6
Source: Nat Commun. 2023 Dec 12;14:8254. doi: 10.1038/s41467-023-44132-y (PMC10716391; doi:10.1038/s41467-023-44132-y)
Supplement: Supplementary file 1 — Supplementary Information [file 41467_2023_44132_MOESM1_ESM.pdf]

Supplementary Information

Unconventional polarization fatigue in van der Waals layered ferroelectric ionic conductor $CuInP_2S_6$

Ziwen Zhou[1,#], Shun Wang[1,#], Zhou Zhou[1], Yiqi Hu[1], Qiankun Li[1], Jinshuo Xue[1], Zhijian Feng[1], Qingyu Yan[1], Zhongshen Luo[1], Yuyan Weng[1,*], Rujun Tang[1], Xiaodong Su[1], Fengang Zheng[1], Kazuki Okamoto[2], Hiroshi Funakubo[2], Lixing Kang[3], Liang Fang[1,*], Lu You[1,*]

[1]School of Physical Science and Technology, Jiangsu Key Laboratory of Thin Films, Soochow University, Suzhou, 215006, China

[2]School of Materials and Chemical Technology, Tokyo Institute of Technology, Yokohama 226-8502, Japan

[3]Division of Advanced Materials, Suzhou Institute of Nano-Tech and Nano-Bionics, Chinese Academy of Sciences, Suzhou 215123, China

*Corresponding authors: lyou@suda.edu.cn; lfang@suda.edu.cn; wengyuyan@suda.edu.cn

[#]These authors contributed equally to this work.

The Supplementary Information includes:

Section 1. A summary of the sample information of the CIPS crystals used in this work

Section 2. Verifications of the anomalous polarization enhancement

Section 3. Morphological evolutions of the CIPS capacitor

Section 4. Frequency-dependent polarization fatigue behaviors

Section 5. Fatigue behavior of CIPS thin flake

Section 6. Compositional study of the fatigued CIPS capacitors

Section 7. The nature of bubble-like protrusions on the fatigued capacitors

Section 8. Depth-profiling XPS study of CIPS capacitors

Section 9. Micro-XRD study

Section 10. PFM study of fatigued CIPS sample

Section 11. Fitting of the AC conductivity

Section 12. Electrode scratching experiment to clarify the polarization enhancement

Table S1. Sample details, device information, and involved experiments.

Table S2. Quantitative atomic concentrations of the marked areas shown in Figure 2a for pristine and fatigued CIPS capacitors.

Fig. S1. Additional experimental results of anomalous polarization enhancement after repetitive electrical cycling.

Fig. S2. Optical images of the fresh and fatigued capacitors.

Fig. S3. Additional optical images of fatigued CIPS capacitors.

Fig. S4. Switching frequency dependent data series.

Fig. S5. Fatigue behavior of CIPS thin flake.

Fig. S6. Elemental distribution of pristine and fatigued capacitor.

Fig. S7. Elemental distribution of the rippled area of the fatigued capacitor.

Fig. S8. Optical images of the front and back sides of the fatigued capacitor.

Fig. S9. Sequential optical images of a CIPS capacitor with the growth of the bubble-like protrusions under electric cycles.

Fig. S10. Compositional investigation inside the bubble-like protrusion.

Fig. S11. Fatigued properties of the sample for XPS study.

Fig. S12. Depth-profiling XPS data of the pristine capacitor presented in stacking plots.

Fig. S13. Depth-profiling XPS data of the fatigued capacitor presented in stacking plots.

Fig. S14. Micro-XRD patterns of pristine and fatigued capacitors.

Fig. S15. Calibration and verification of quantitative PFM.

Fig. S16. Quantitative PFM imaging of pristine and fatigue capacitors.

Fig. S17. AC conductivity fitting for pristine and fatigued CIPS.

Fig. S18. Electrode scratching experiments.

Fig. S19. Fatigue test under vacuum at room temperature.

Section 1. A summary of the sample information of the CIPS crystals used in this work

Table S1. Sample details, device information, and involved experiments.

Crystal No.	Thickness	Electrode size	Coercive field @ 100 Hz	Involved experiments
#1	≈ 9.2 μm	200 * 200 μm^2 squares / 300-μm-diameter circular pads	≈ 29.1 kV/cm	Field dependence (Fig. 1); Cross-sectional Raman (Fig. 5); Dielectric spectroscopy (Fig. 6)
#2	≈ 10.5 μm	300-μm-diameter circular pads	≈ 28.2 kV/cm	Frequency dependence (Fig. S4); temperature dependence (Fig. 7)
#3	≈ 16.6 μm	200 * 200 μm^2 squares	≈ 25.3 kV/cm	EDS (Fig. 2);Correlated microscopic imaging (Fig. 3); Fig. S1c-f; Fig. S2
#4	≈ 15.0 μm	200 * 200 μm^2 squares	≈ 26.7 kV/cm	XPS (Fig. 4); micro-XRD (Fig. S14); quantitative PFM (Fig. S16); Electrode cutting (Fig. S18)

Section 2. Verifications of the anomalous polarization enhancement

As described in the main text, the polarization usually shows an initial enhancement under electric cycling. This effect is more prominent when small field and low frequency pulse (**Fig. S1c, d**) was used, in which case the remanent polarization can be even doubled. To confirm the measured polarization is not due to leakage or other non-remanent contributions, remanent hysteresis measurement was employed to subtract the non-remanent components from the total measured polarization (**Fig. S1a, b**). Besides, the switchable remanent polarization was also measured by the field-dependent PUND method (**Fig. S1e, f**), which confirms the intrinsic nature of the enhanced remanent polarization. However, we also noticed that the incremental part of the polarization usually requires higher electric field to saturate, indicating large switching barrier for this extra polarization.

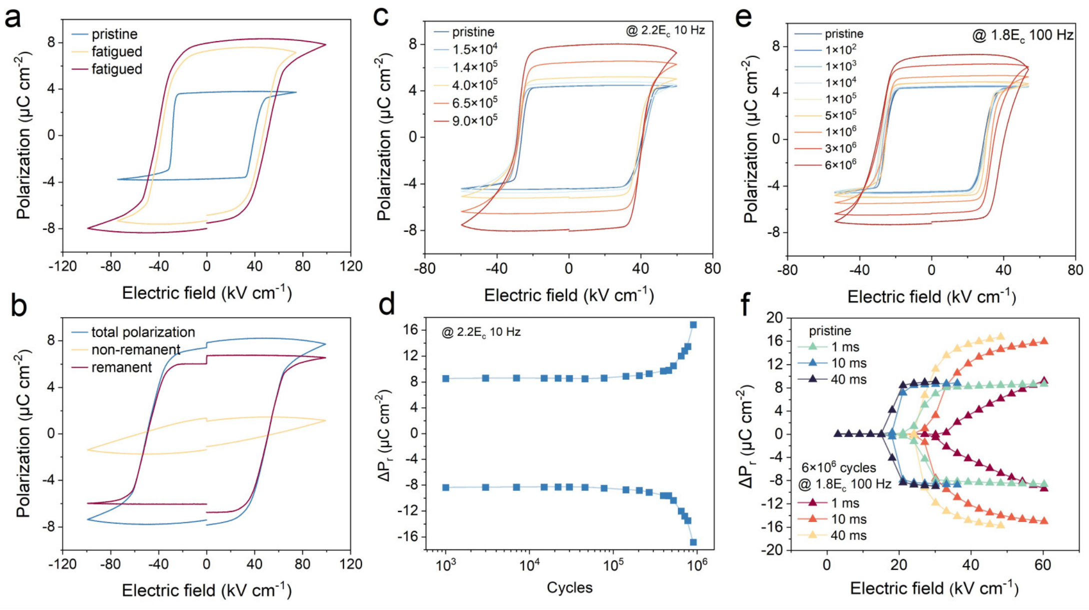

Fig. S1. Additional experimental results of anomalous polarization enhancement after repetitive electrical cycling. (**a**) Polarization hysteresis loops and (**b**) remanent polarization hysteresis loop of the CIPS capacitor fatigued under 1.7E_C at 100 Hz as described in the main text. (**c**) Evolution of polarization hysteresis loop and (**d**) switchable remanent polarization with different switching cycles under an electric field of 2.2E_C and a frequency of 10 Hz. (**e**) Evolution of polarization hysteresis loop with cumulative switching cycles under an electric field of 1.8E_C and a frequency of 100 Hz. (**f**) Switchable remanent polarization as a function of switching bias for the same capacitor in (e) before and after fatigue.

Section 3. Morphological evolutions of the CIPS capacitors

The surface morphology of the CIPS capacitor dramatically changes under repetitive switching cycles. The example shown in **Fig. S2** is a CIPS capacitor cycled by low electric field. Dense bubbles were formed under the electrode and very fine surface ripples were created in the surrounding area. These surface features strongly scatter the incident light, resulting in bright region and dark region in dark-field and transmission imaging modes, respectively.

Fig. S3 compares the morphology evolutions under different electric fields. Usually, higher electric field tends to produce much larger surface wrinkles, which in some cases even cause a complete delamination of vdW layers and a total loss of measurable polarization.

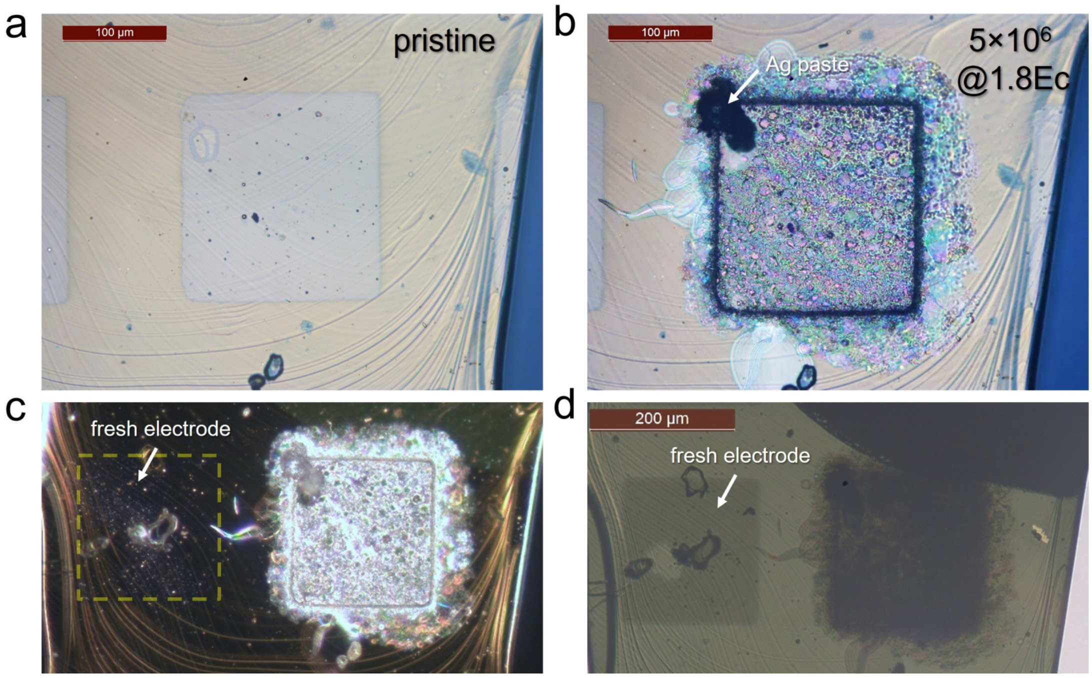

Fig. S2. Optical images of the fresh and fatigued capacitors. (**a**) Bright-field optical image of the pristine CIPS capacitor. (**b**) Bright-field image of the same capacitor after 5×10^6 cycles of repetitive switching at $1.8E_C$. (**c**) The dark-field optical image showing a fresh capacitor and the fatigued one. (**d**) The transmission optical image of a fresh capacitor and the fatigued one.

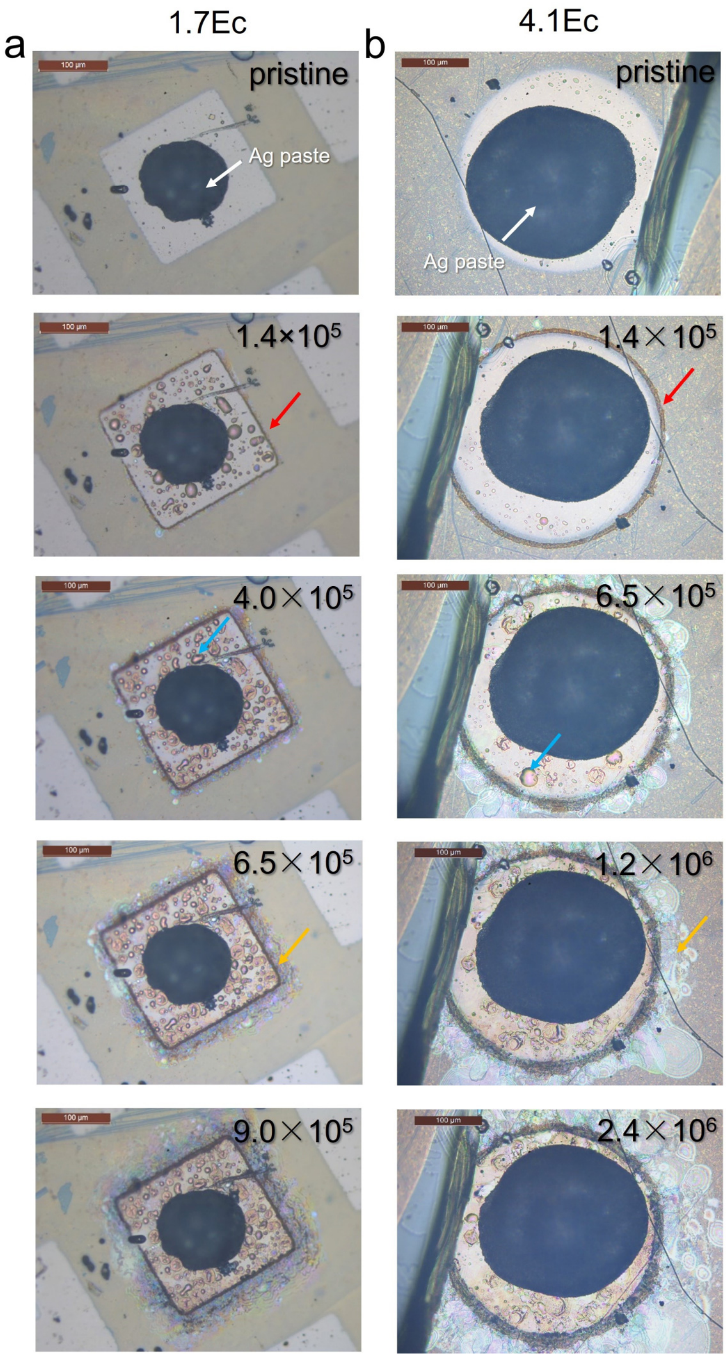

Fig. S3. Additional optical images of fatigued CIPS capacitors. Sequential optical images of the CIPS capacitors after cumulative polarization switching cycles under an electric field of (**a**) 1.7E_C and (**b**) 4.1E_C.

Section 4. Frequency-dependent polarization fatigue behaviors

The measurements of switching frequency dependence in **Fig. S4** indicate that cycling using low frequency tends to induce the polarization enhancement, while under higher frequencies no clear trend for the polarization fatigue can be concluded. It seems like the polarization degradation is closed related to the increased leakage of the capacitors, and the leakage path formation in the sample is probably stochastic and erratic. Nevertheless, all the samples measured still show a polarization upturn before the final degradation.

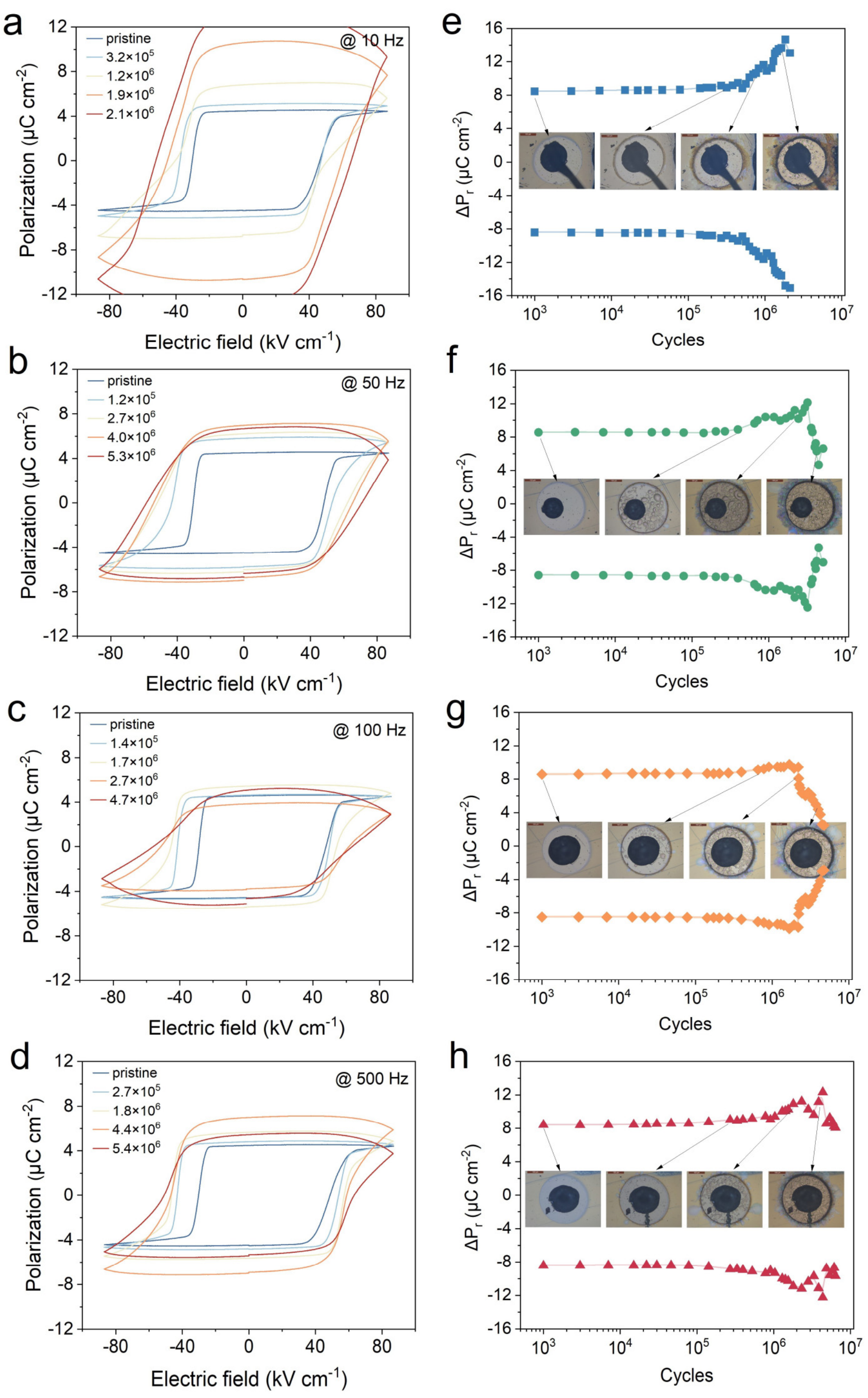

Fig. S4. Switching frequency dependent data series. (**a-d**) Evolutions of the polarization hysteresis loops of CIPS capacitors after cumulative cycles with a frequency of 10, 50, 100, and 500 Hz, respectively. The switching field and pulse width is fixed at $2.8E_C$ and 1 ms, respectively. (**e-h**) Switchable remanent polarization as a function of the switching cycles with a frequency of 10, 50, 100, and 500 Hz, respectively. The insets are the corresponding optical images of the capacitors recorded at different cycling stages as denoted by the arrows.

Section 5. Fatigue behavior of CIPS thin flake

We also tried to investigate the fatigue property of CIPS thin flakes. However, there are currently two difficulties that prevent us from measuring few-layer samples. First, the capacitor becomes too leaky for polarization hysteresis loop measurement below 100 nm, and secondly, the area of the exfoliated few-layer flake is usually too small (less than 10*10 μm^2) for a reliable measurement of the switched polarization. Nevertheless, we still managed to conduct fatigue study on a 172-nm-thick thin flake of CIPS as shown in **Fig. S5**. The fresh capacitor shows well-defined polarization hysteresis loop with a remanent polarization of ≈3.5 μC cm^{-2}. The coercive field is on the order of 100 kV cm^{-1}, which is much larger than that of the bulk and consistent with the semi-empirical Kay-Dunn law for ferroelectrics. With increasing cycles, the remanent polarization of the thin flake displays similar enhancement behavior to the bulk counterpart, followed by a sudden breakdown near 10^6 cycles. Based on the preliminary results, we didn’t find obvious difference of the fatigue behavior in hundred-nanometer-thick flakes. Certainly, further studies on thinner flakes are required to verify the possible size effect.

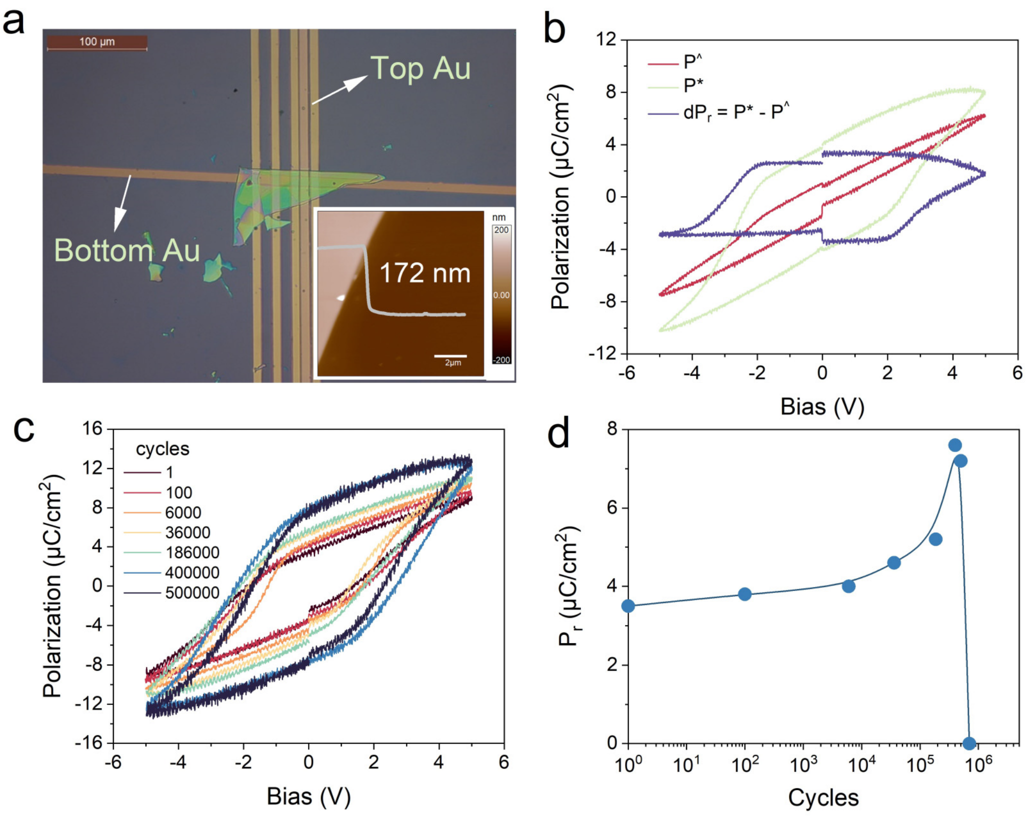

Fig. S5. Fatigue behavior of CIPS thin flake. (**a**) Optical image of the cross-bar capacitor of a CIPS thin flake. The inset shows the AFM topographic image to determine the flake thickness. (**b**) Remanent hysteresis loop of the thin flake capacitor by subtracting the non-remanent contribution of the polarization. (**c**) Polarization hysteresis loops of the thin flake capacitor after cumulative switching cycles. (**d**) Remanent polarization as a function of the switched cycles.

Section 6. Compositional study of the fatigued CIPS capacitors

EDS is generally considered as a semi-quantitative elemental analysis technique. However, since the measurement conditions are fixed among different samples, we found the results highly reproducible and thus reliable. As shown in **Table S2**, the average composition (the box areas in Figure 2a of the main text) of the fatigued capacitor shows a much higher oxygen abundance compared to that of the pristine one. However, the relative atomic ratios between Cu, In, P and S remain almost the same, as can be seen in the Cu/In ratios of the fatigued and pristine samples.

The EDS maps of four constituent elements of CIPS are shown in **Fig. S6**. The seeming decrease of the other four elements in the fatigued capacitor is because of the inclusion of oxygen in atomic percentage calculation for the elemental maps. The line profiles of each element are presented in **Figure 2d**, revealing the accumulation of Cu at the electrode edge compared to the other three elements.

Fig. S7 shows the comparison of the element distribution between the rippled and pristine area. Apparently, there is significant enrichment of oxygen in the rippled area. For other elements, because of the overwhelming contribution from the underneath bulk material (depth ≈1 μm) in EDS measurements, it is difficult for us to discern the small composition variations on the surface ripples. Nevertheless, by enhancing the contrast of the EDS map, we were able to observe the difference: there is slight Cu enrichment in the rippled area compared to the other three elements, in agreement with the result found in **Figure 2d**.

Table S2. Quantitative atomic concentrations of the marked areas shown in Figure 2a for pristine and fatigued CIPS capacitors.

Element	Pristine-1 (atom %)	Pristine-2 (atom %)	Pristine-3 (atom %)	Fatigued-1 (atom %)	Fatigued-2 (atom %)	Fatigued-3 (atom %)
O	6.17	7.33	6.74	24.68	25.28	23.74
Cu	10.22	10.23	10.24	8.57	8.53	9.00
In	8.91	8.71	8.86	7.42	7.67	7.46
P	21.29	21.04	21.09	17.50	17.41	17.63
S	53.41	52.68	53.07	41.83	41.11	42.17
Cu/In ratio	1.15	1.17	1.16	1.15	1.11	1.20

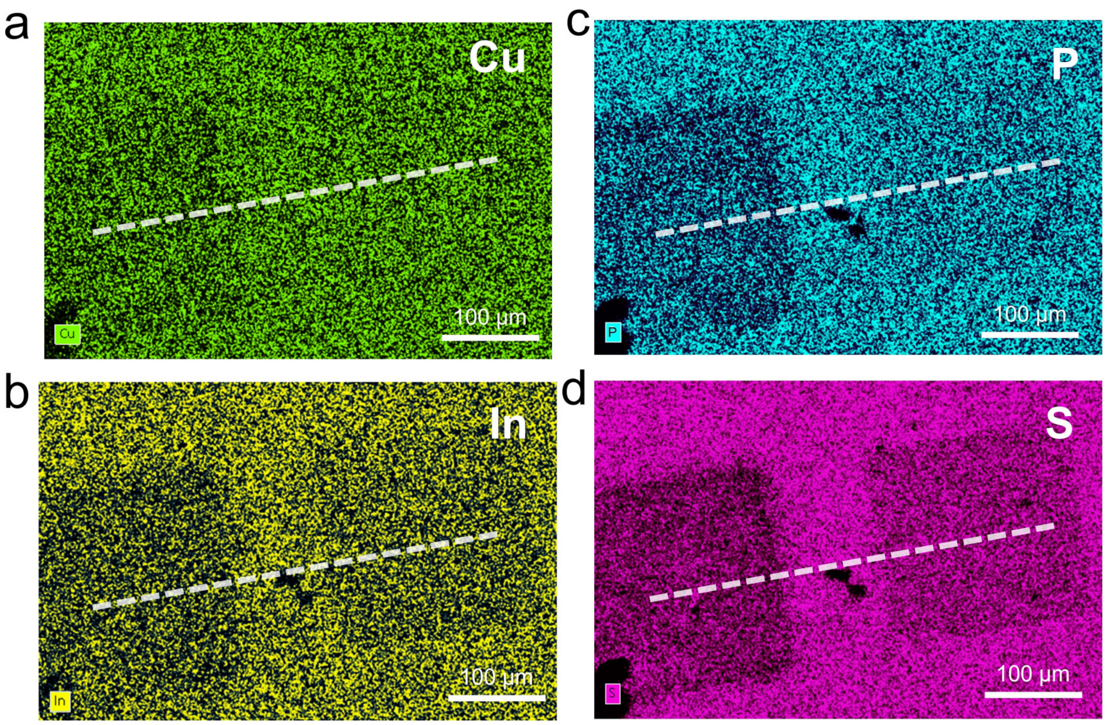

Fig. S6. Elemental distribution of pristine and fatigued capacitor. (**a**) Cu, (**b**) In, (**c**) P, and (**d**) S distribution maps of **Figure 2a**. The corresponding line profiles of each element were plotted in **Figure 2d**.

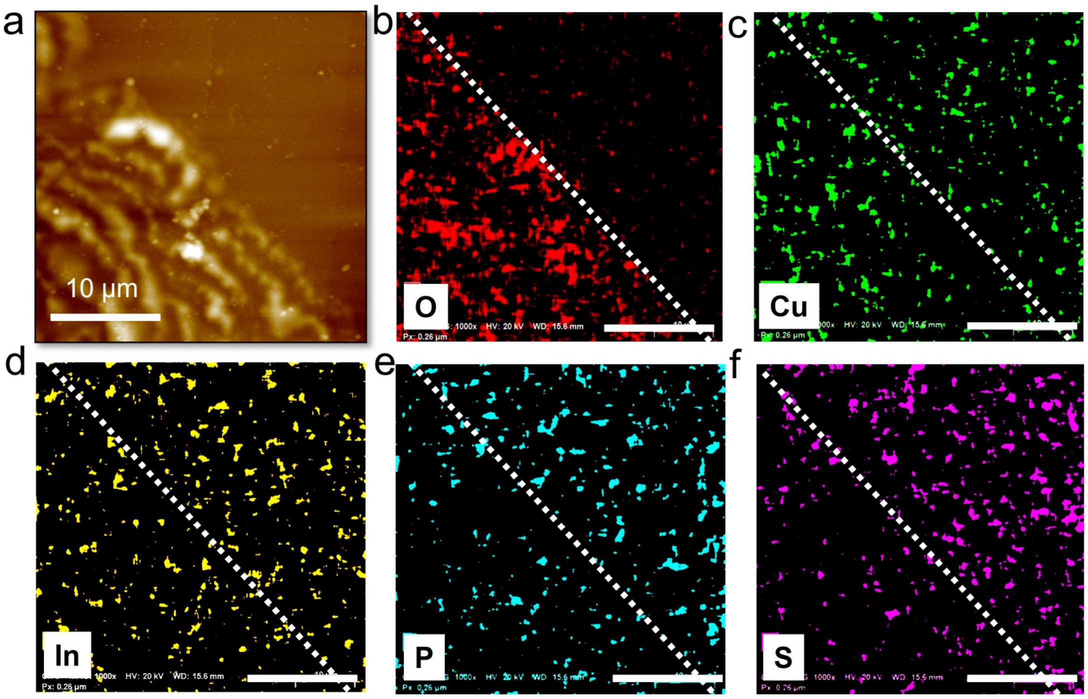

Fig. S7. Elemental distribution of the rippled area of the fatigued capacitor. (**a**) AFM topographic image and (**b-f**) corresponding EDS elemental maps of the rippled area of the fatigued capacitor. (b) O, (c) Cu, (d) In, (e) P, and (f) S map.

Section 7. The nature of bubble-like protrusions on the fatigued capacitors

In **Fig. S8a**, the silver paste on the capacitor is used to improve the reliability of the electric contact during long-time fatigue tests. Occasionally, the silver paste can be removed by the metal probe without damaging the device. Unexpectedly, we found the electrode area underneath the paste remained flat and clean, without the formation of the protrusions (**Fig. S8b**). Additionally, we also found that the bottom electrode of the capacitor contains much less and smaller protrusions than the front side (**Fig. S8c, d**). More interestingly, the protrusions behaved like a balloon showing repetitive inflations and evacuations without breaking up, as indicated by the blue arrows in **Fig. S9**. These findings made us to speculate that the surface protrusion is not consisted of solid or liquid matter originating from the CIPS degradation.

To unravel the nature of the protrusion, we used micro-sized tungsten probe to scratch and open up the bubble (**Fig. S10a-c**). The whole process is recorded in **Supplementary video 2**. Apparently, the bubble is filled by gaseous matter, which is contained by the impermeable gold film. The exposed CIPS surface shows dark rings, predominantly at the edge of the bubble (**Fig. S10d**). Subsequent Raman and EDS spectra confirm that the dark area inside the bubble also possesses oxygen enrichment and the characteristic peak of S_8 rings, suggesting similar surface oxidation as the electrode edge and rippled area (**Fig. S10e-k**). Combining all the experimental evidence above, we can conclude that the substance inside the protrusion is simply air, which is pumped into the bubble due to the large repetitive peizostrain during electric cycling. The potential air transport pathway is through the electrode-sample interface due to their weak vdW interaction.

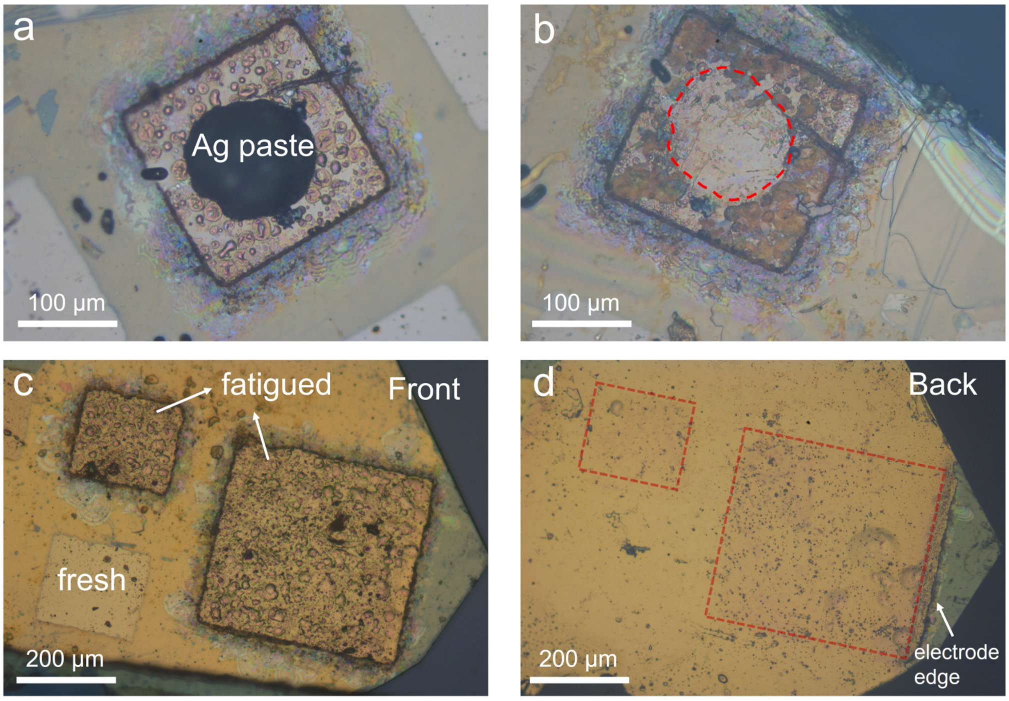

Fig. S8. Optical images of the front and back sides of the fatigued capacitor. Optical images of (**a**) a fatigued capacitor and (**b**) after removing the Ag paste on the surface. Optical images of the (**c**) front and (**d**) back sides of the fatigued capacitors.

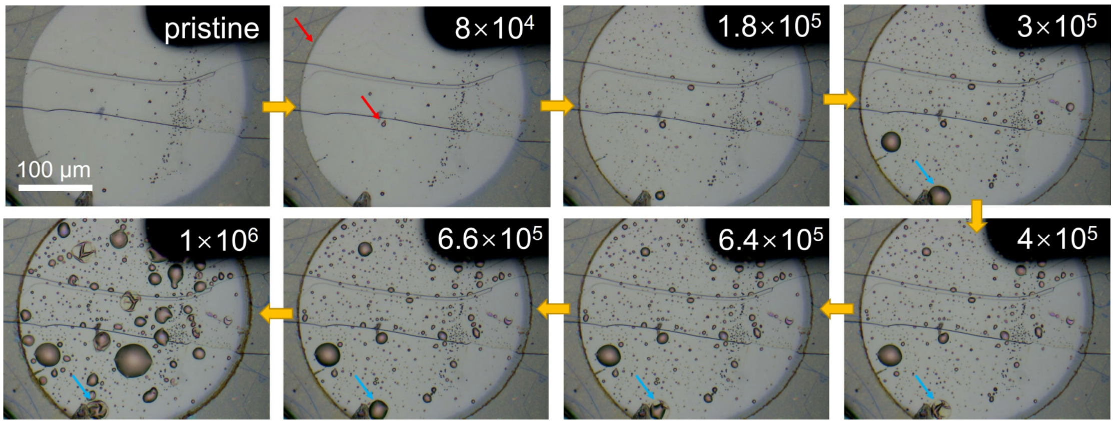

Fig. S9. Sequential optical images of a CIPS capacitor with the growth of the bubble-like protrusions under electric cycles. The red arrows indicate the concurrence of dark electrode edge and the bubble protrusion. The blue arrows highlight the inflation and evacuation of the bubble-like electrode membrane.

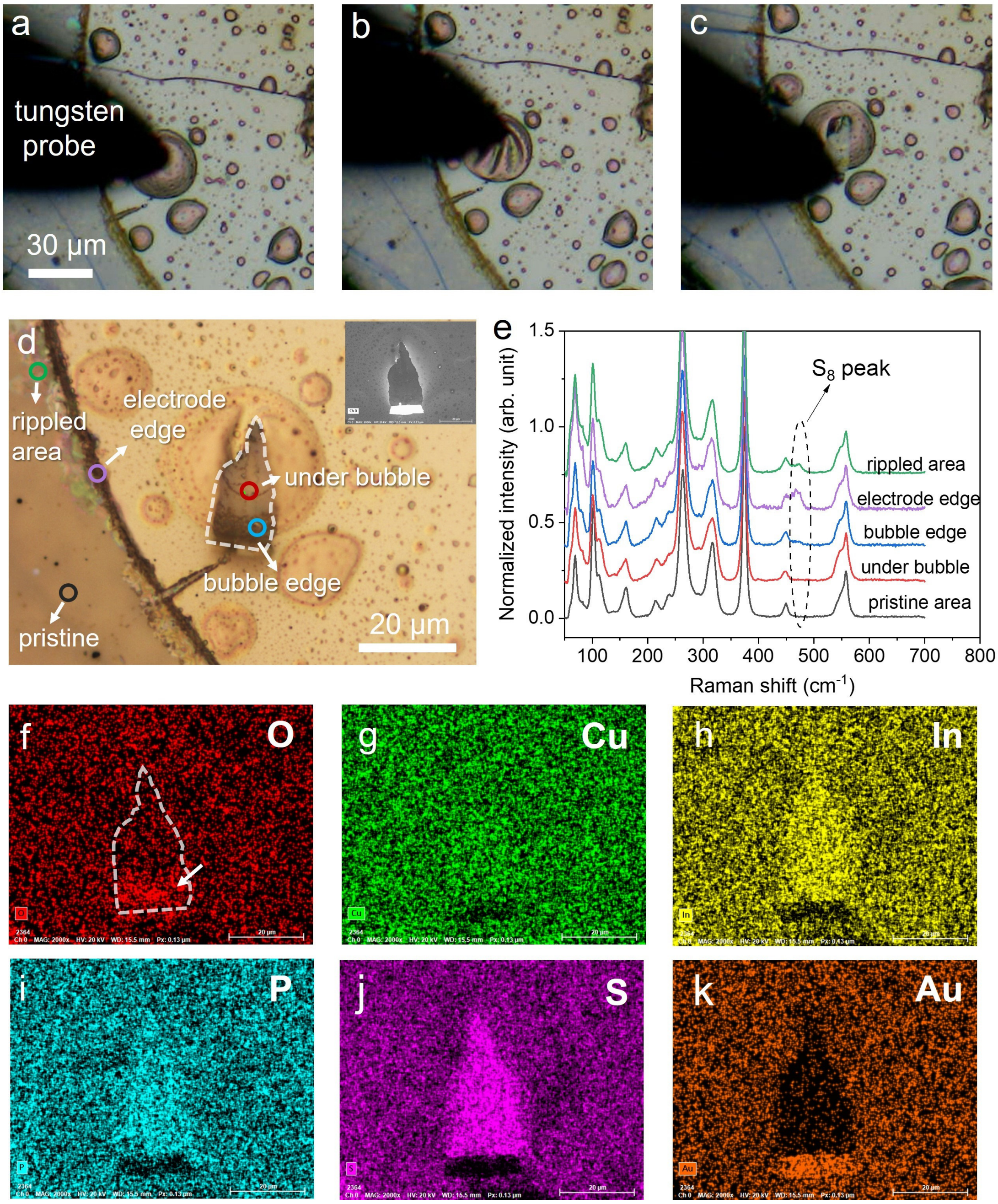

Fig. S10. Compositional investigation inside the bubble-like protrusion. (**a-c**) Sequential optical images showing the process of opening up a surface bubble using a micromanipulator probe. (**d**) The optical image of a bubble-like protrusion with its interior exposed. The inset is the corresponding SEM image. (**e**) Corresponding Raman spectra of selected spots as denoted by the colour circles in (d). EDS maps of different elements: (**f**) O, (**g**) Cu, (**h**) In, (**i**) P, (**j**) S, and (**k**) Au.

Section 8. Depth-profiling XPS study of CIPS capacitors

We conducted depth-profiling XPS on both pristine and fatigued capacitors to reveal more chemistry information along the sample depth. The sample details are shown in **Fig. S11**. The probed areas were located at the center of the capacitors with an X-ray beam size of 50 μm in diameter. The depth profiling was performed by sequential Ar^+ ion etching, in which the nominal thickness is estimated based on the etching rate of SiO_2. By continuously monitoring the S 2p signal, it started to appear after a nominal etching depth of 15 nm, which is comparable to the thickness of the Au electrode.

To check the possible changes in element valence states, we compared the depth-resolved XPS spectra in stacking plots as shown in **Fig. S12** and **Fig. S13**. For the pristine CIPS, there is no peak shift for all elements, confirming the uniform chemical valence in the sample. As for the fatigued sample, we didn't observe significant peak shifts or additional peaks in the XPS depth-profiling spectra of Cu 2*p*, In 3*d* and S 2*p*. In contrast, the P 2*p* spectra contains the characteristic peak corresponding to the P-O_x bond, whose intensity gradually diminishes in layers far from the surface. In the meantime, the peak attributed to P-S gradually grows up. This finding is in accordance with the oxygen concentration along the depth, suggesting the oxidation of phosphorus in the surface layer of CIPS. However, we cannot exclude possible oxidation of Cu on the surface, because the binding energies of Cu^I-O and Cu^I-S bonds are very similar to each other.

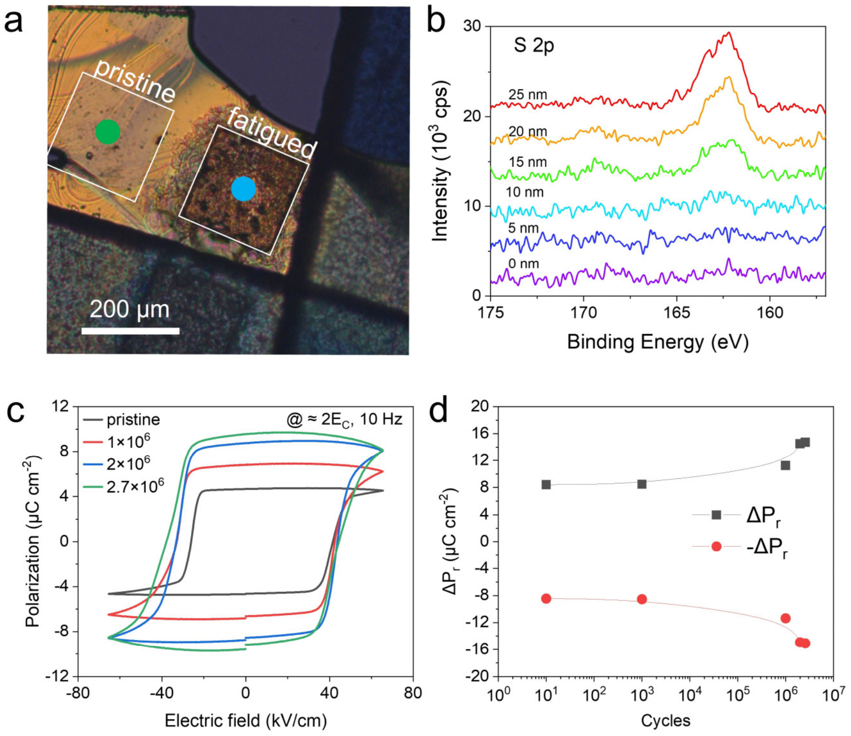

Fig. S11. Fatigued properties of the sample for XPS study. (**a**) Optical image of the CIPS crystal with pristine and fatigued capacitors. The color dots denote the probed areas (beam diameter ≈ 50 μm). (**b**) S 2*p* spectra recorded at nominal etched depths. (**c**) Polarization hysteresis loops of the capacitor after different switching cycles. (**d**) Switchable remanent polarization as a function of the electrical cycles.

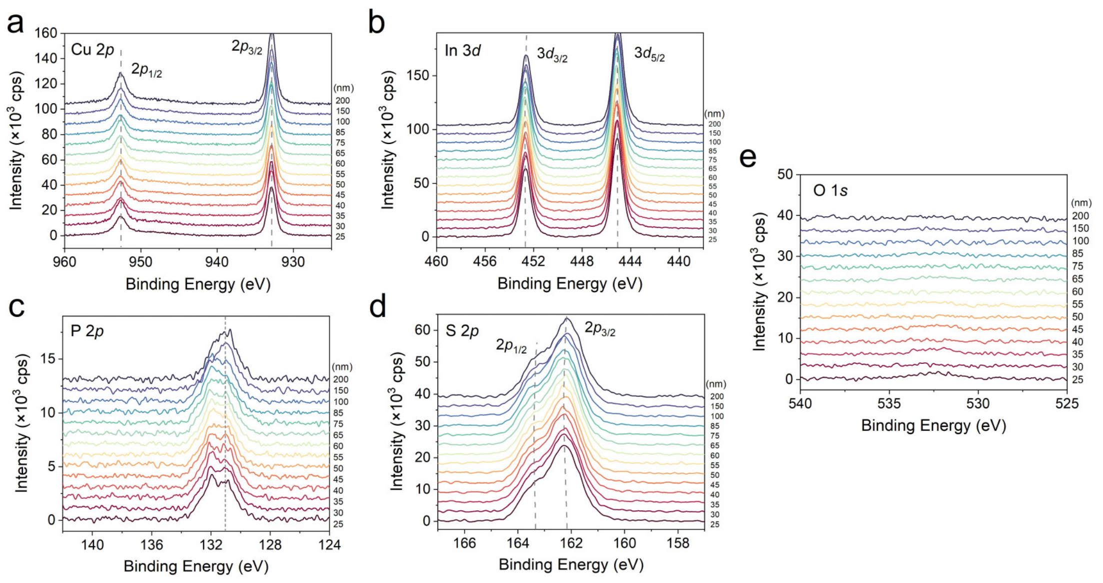

Fig. S12. Depth-profiling XPS data of the pristine capacitor presented in stacking plots. (**a**) Cu 2*p*, (**b**) In 3*d*, (**c**) P 2*p*, (**d**) S 2*p*, and (**e**) O 1*s* spectra.

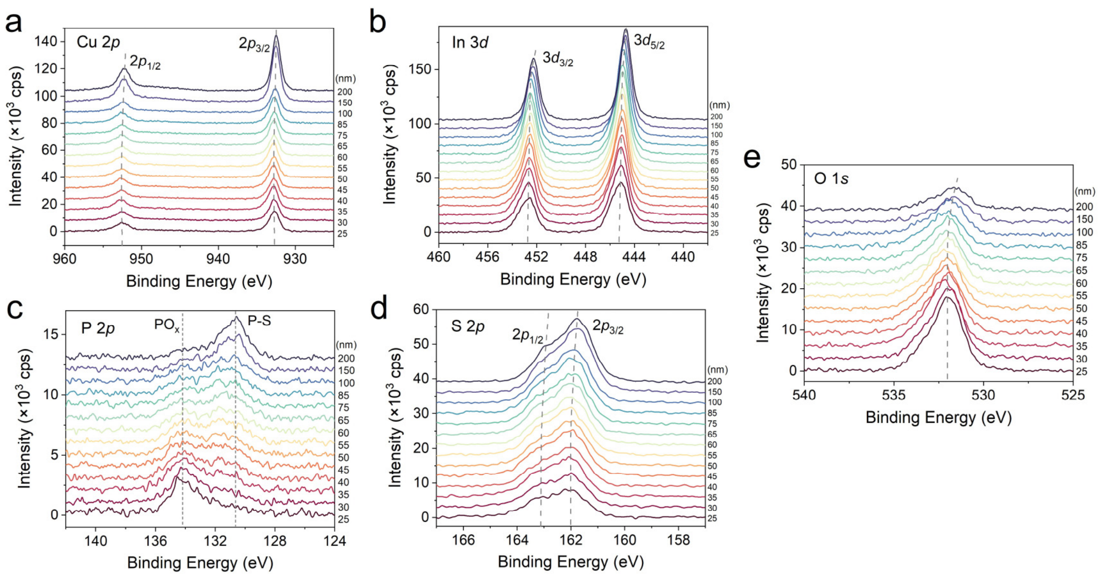

Fig. S13. Depth-profiling XPS data of the fatigued capacitor presented in stacking plots. (**a**) Cu 2*p*, (**b**) In 3*d*, (**c**) P 2*p*, (**d**) S 2*p*, and (**e**) O 1*s* spectra.

Section 9. Micro-XRD study

To gain more insight from the lattice structure of fatigued sample, we have performed Micro X-ray Diffraction (μXRD). The incident X-ray was focused to a beam diameter of 50 μm to detect the structural changes due to fatigue test within an electrode. As shown in **Fig. S14**, the 2D detector allows us to record the conventional 2θ-θ scan and rocking curve along χ direction. In the 2θ-θ pattern, the peak intensities of the fatigued capacitor are reduced compared to the pristine one. However, we didn't observe noticeable peak shift induced by fatiguing, which rules out the formation of high-polarization phase in the fatigued capacitor[1]. In the χ scan, the pattern of the fatigued sample includes a sharp central peak and a diffused background, which can be attributed to the rippling of the vdW layers.

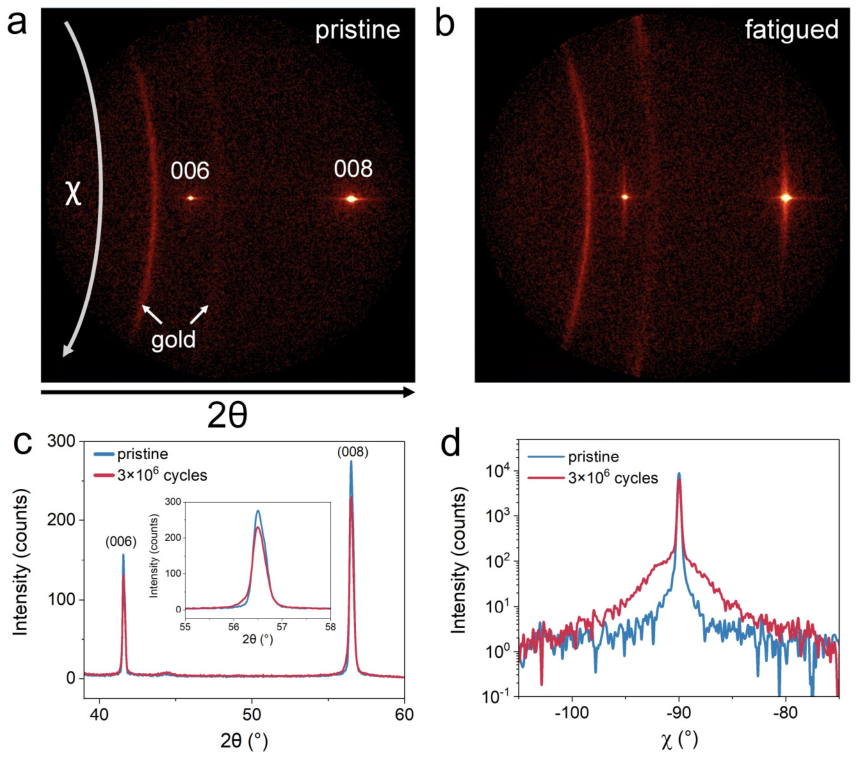

Fig. S14. Micro-XRD patterns of pristine and fatigued capacitors. 2D XRD maps of (a) pristine and (b) fatigued CIPS capacitors around (006) and (008) Bragg peaks. (c) 2θ-θ scan of the (006) and (008) peaks of the CIPS capacitors. The inset is the zoomed-in plot of the (008) peak. (d) χ scan of the (008) peak of the CIPS capacitors.

Section 10. PFM study of fatigued CIPS sample

To minimize the electrostatic contributions, our protocol in dual AC resonance tracking (DART) PFM measurement is to check the surface potential difference between tip and sample by Kelvin probe force microscopy (KPFM) first, and make sure their potential difference is below 1 V. Stiff probes with large spring constants are widely reported to effectively reduce the electrostatic effect[2, 3]. Therefore, we carried out quantitative PFM under both resonant-enhanced mode (DART) using a standard probe (k ≈ 2 N/m) and non-resonant mode using a stiff probe (k ≈ 40 N/m) for cross-checking. For both methods, the values of invOLS were carefully calibrated using contact force curve beforehand, which translates the AC deflection signal (volt) into the actual displacement (nm) as detected by the probe. In DART method, the measured PFM signals (amplitude, phase, resonant frequency) were fitted using the simple harmonic oscillator (SHO) model, from which the quantitative piezoresponse amplitude can be derived[4]. In non-resonant mode, driving frequency far from the resonance was chosen to prevent signal enhancement (**Fig. S15d, e**). The obtained quantitative amplitude images using the two different methods are in good agreement with each other (**Fig. S15a-c**), confirming the reliability of our results.

Based on the method established above, we re-measured the effective piezoresponse of the fresh and fatigued capacitors using quantitative DART-PFM with SHO fitting, as shown in **Fig. S16**. The capacitors were poled into single polarization state first, followed by the exfoliation of the top electrodes to facilitate the PFM imaging. The fresh capacitor exhibits uniform piezoresponse across the scanned area of 30*30 μm^2, suggesting the high quality of our single crystal sample. In comparison, the fatigued capacitor with enhanced polarization contains regions with suppressed piezoresponse. Besides, scattered small domains with opposite phase signal can be observed. There are two possible explanations for these small domains. It could be the frozen domains, which are not switchable during the pre-poling process. Or, it can be interpreted as the high-polarization phase with positive d_{33}, and consequently opposite phase signal[1]. With only the PFM results, it is not possible for us to distinguish these two scenarios. However, our micro-XRD (**Fig. S14**) results completely ruled out the existence of the high-polarization phase in the fatigued capacitor. Hence, the low-piezoresponse domains are more likely frozen domains or domains with glassy dipoles, which contribute to the dielectric relaxation in the permittivity spectrum.

Majority of the fatigued capacitor shows a piezoresponse comparable to that of the fresh one. By quantitatively measuring the piezoresponse using a stiff probe under non-resonant mode (**Fig. S15e, f**), we obtained the effective d_{33} of the fresh and fatigue sample to be ≈ 20 pm/V, a value much smaller than that reported for CIPS bulk. This is because in current study, the

piezoresponse is measured without top electrode. Therefore, the AFM tip only excites a small volume of the sample, which is subjected to strong clamping by the surrounding bulk.

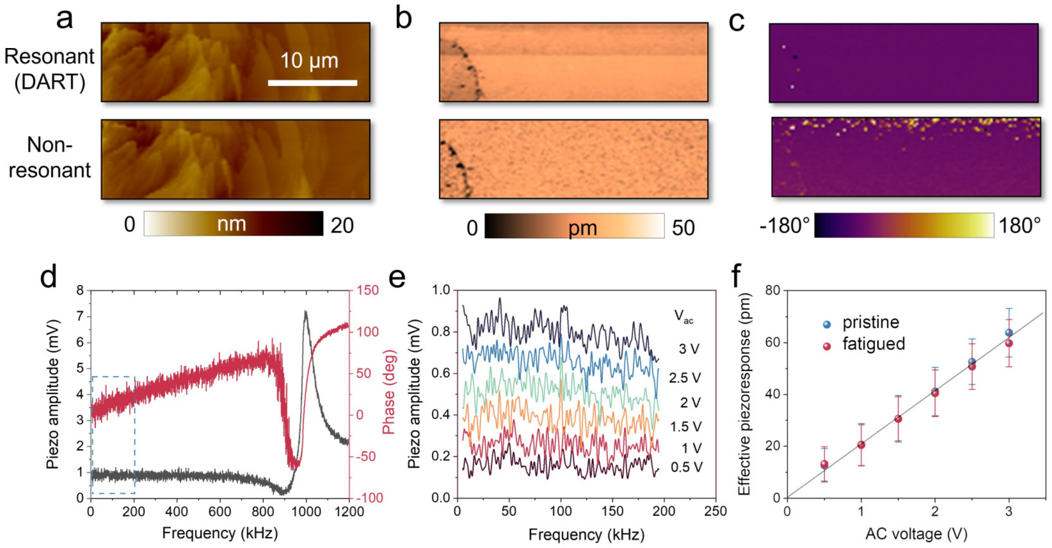

Fig. S15. Calibration and verification of quantitative PFM. (**a**) Topographic, (**b**) quantitative PFM amplitude, (**c**) PFM phase images of CIPS sample under resonant (upper panel, at 300 kHz) and non-resonant (bottom panel, at 30 kHz) modes. (**d**) Frequency-dependent piezoresponse amplitude (AC deflection) and phase measured by a stiff probe (k ≈ 40 N/m). (**e**) Low-frequency piezoresponse amplitude of a fresh CIPS capacitor as a function of the AC driving voltage. (**f**) Effective piezoresponse as derived from (**e**) under different AC voltages. The error bars denote the standard deviations of the data points.

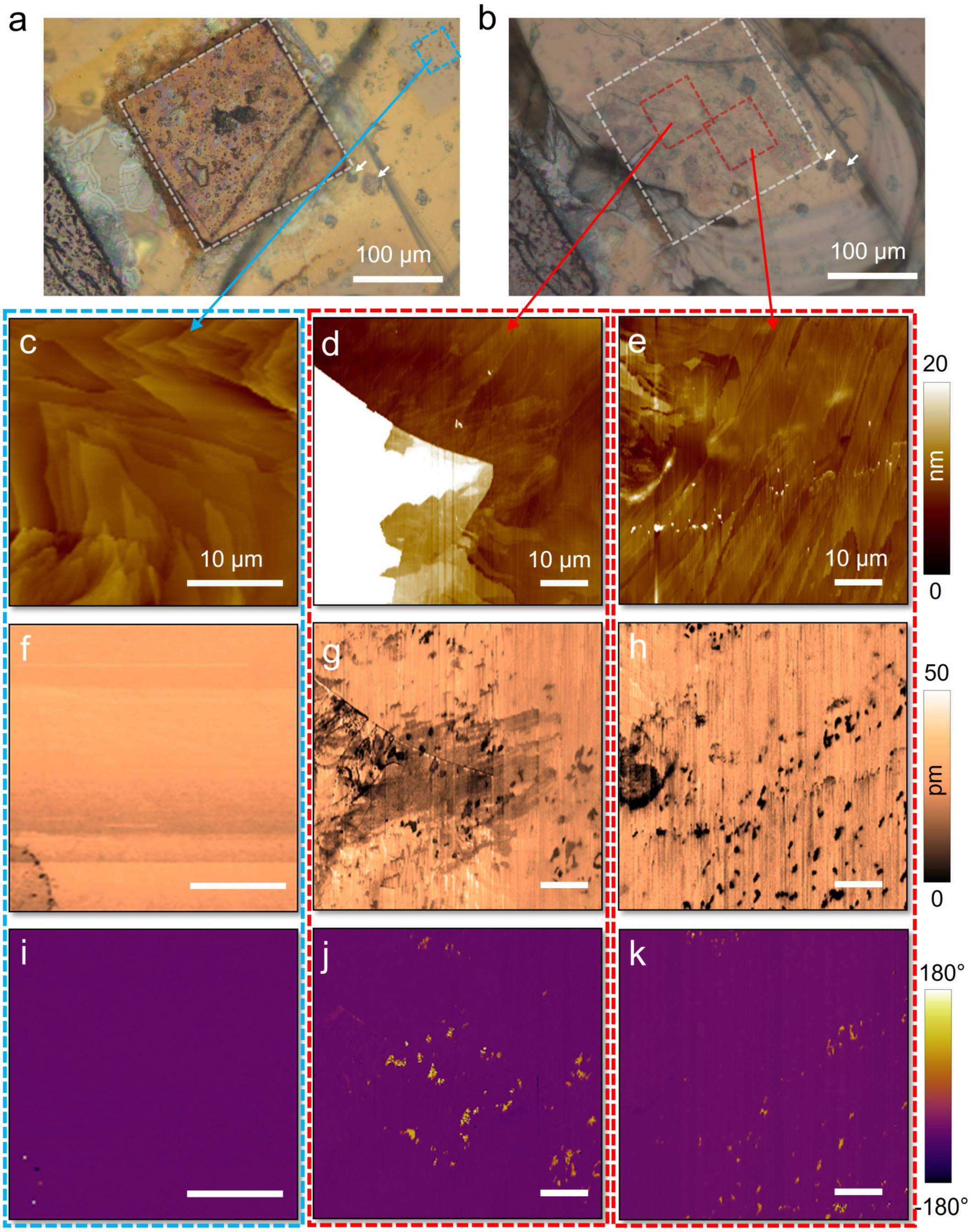

Fig. S16. Quantitative PFM imaging of pristine and fatigue capacitors. Optical images of the CIPS single crystal (**a**) before and (**b**) after electrode exfoliation. The fresh and fatigued capacitors were poled into single polarization state before the exfoliation. The blue and red boxes indicate the scan areas for fresh and fatigued capacitors, respectively. The white arrows denote markers for positioning. (**c-e**) Topographic, (**f-h**) PFM amplitude, and (**i-k**) PFM phase images of the fresh and fatigued areas as indicated by corresponding color boxes. The PFM was performed in DART mode using a PtIr-coated probe (k ≈ 2 N/m). The piezoresponse amplitude was fitted using SHO model to obtain quantitative values.

Section 11. Fitting of the AC conductivity

The temperature-dependent AC conductivities of the CIPS samples are calculated based on the imaginary part of electric permittivity ε_r'' using the following equation, provided the ionic space charge is the dominant factor for the dielectric dispersion,

$$\sigma = 2\pi f \varepsilon_0 \varepsilon_r'' \quad (1)$$

where f is the measurement frequency and ε_0 is the vacuum permittivity. Because of the applicable condition for equation (1), only those data at relatively high but below phase transition temperature are used. The AC conductivity is then plotted as a function of frequency in Figure S11 for different temperatures. Next, the data are fitted by Jonscher's power law using the following equation[5],

$$\sigma = \sigma_{dc} + A(2\pi f)^s \quad (2)$$

where σ_{dc} is the conductivity at DC limit, A is the pre-exponential constant, and s is the power law exponent with $0 < s < 1$.

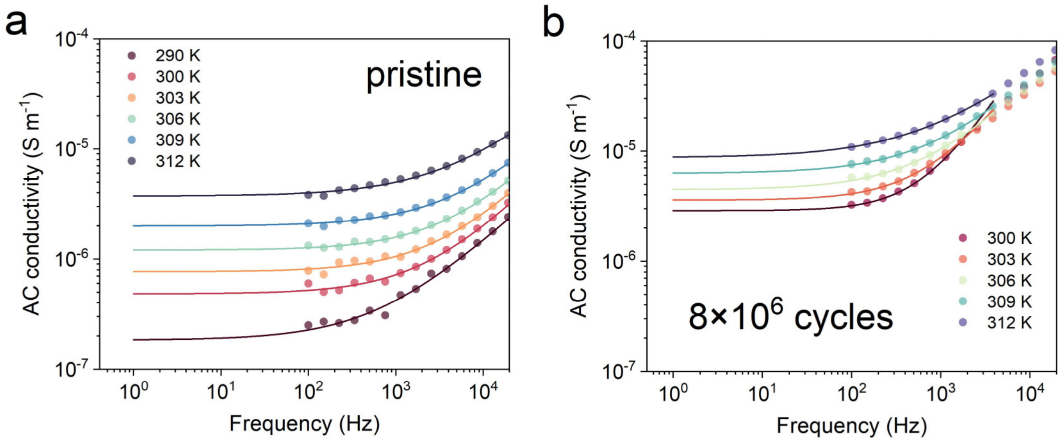

Fig. S17. AC conductivity fitting for pristine and fatigued CIPS. Temperature-dependent AC conductivity of (**a**) pristine and (**b**) fatigued CIPS capacitor as derived from the dielectric spectra. The data are fitted by Jonscher's power law as denoted by the solid lines.

Section 12. Electrode scratching experiment to clarify the polarization enhancement

In the μXRD study, we have already excluded that the anomalous polarization enhancement is due to the formation of high-polarization phase. Moreover, in the correlated microscopic imaging studies, we also confirmed substantial surface oxidation at the electrode edge and its surrounding areas. The oxidized surface layer possesses enhanced electrical conductivity, which may potentially increase the electrode area and the measured polarization.

To confirm our hypothesis, we conduct a set of experiments by scratching the top electrode using micro-manipulator probe station to re-define the actual electrode area. As shown in **Fig. S18**, we chose a fresh capacitor, measured its electrode area and polarization charge (**Fig. S18a**). Then, we cycled the capacitor using an electric field of $2E_c$ for 1.3×10^6 cycles, and re-measured the hysteresis loop. As expected, the switchable polarization charge was greatly increased. This time, we also estimated the total area of the capacitor including the surrounding oxidized area (**Fig. S18b**). Next, we scratched the sample surface using tungsten probe (10 μm diameter) to isolate the central capacitor from the conductive oxidized area (**Fig. S18c**). Since the oxidation is mainly confined in the surface layer less than 200 nm depth, we could easily scratch away the oxidized layer to expose a fresh CIPS surface, which is insulating. We then re-measured the polarization hysteresis loop of the scratched capacitor, and found its switchable polarization charge reduced back to the original value! The result unambiguously suggests the enhancement of the polarization originated from an increase in the actual electrode area due to the formation of the conductive surface layer after electric cycling. For further confirmation, we re-defined a smaller capacitor by scratching (**Fig. S18d**) and measured its switchable polarization. By normalizing the total measured charges to the actual device areas, we found the polarization values are similar for these four cases, thus confirming our argument.

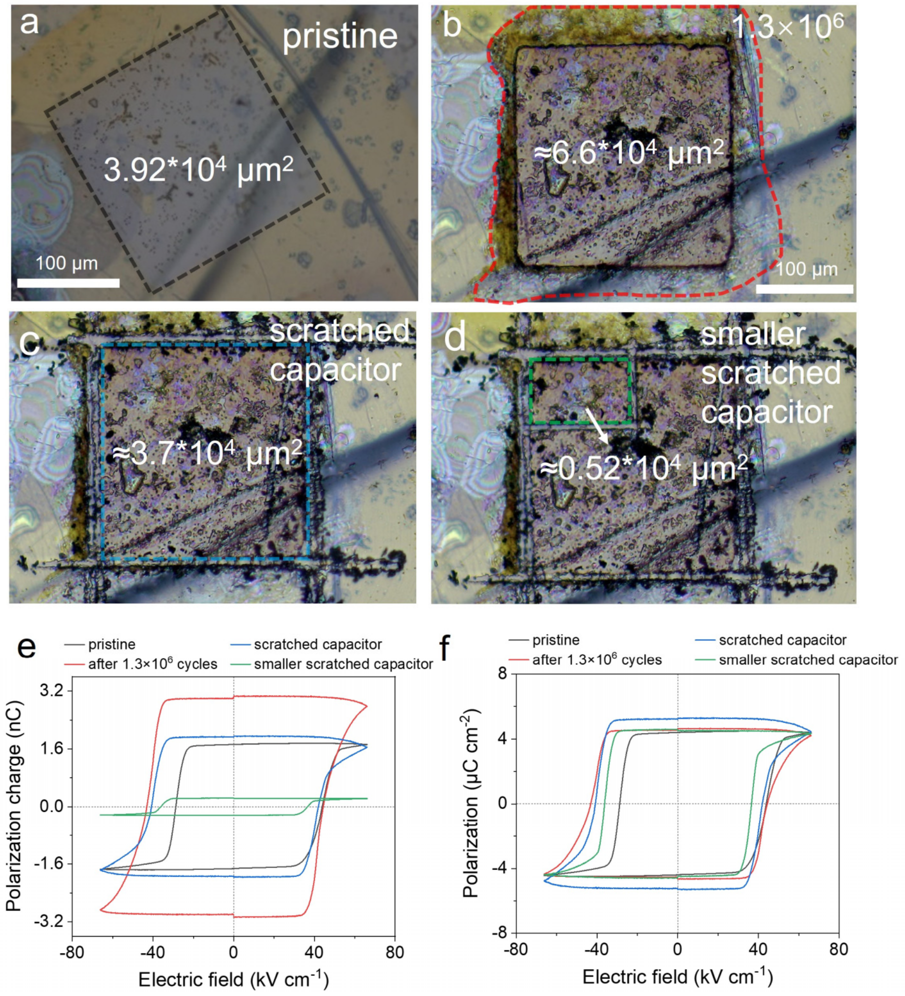

Fig. S18. Electrode scratching experiments. Optical images of (**a**) pristine capacitor, (**b**) fatigued after 1.3×10^6 cycles, (**c**) after scratched and isolated from the surrounding conductive area, and (**d**) after creating a smaller isolated capacitor by scratching. (**e**) Measured total polarization charge hysteresis loops in the four cases of (a-d). (**f**) Recalculated polarization values after normalizing to the actual electrode areas.

Section 13. Fatigue test under vacuum at room temperature

To verify the oxidation effect on the fatigue behavior of CIPS, we have also conducted fatigue test under vacuum, yet at room temperature to retain the ionic conductivity. As shown in **Fig. S19**, the switchable polarization decreases only slightly up to 5.5×10^7 cycles, and the hysteresis loops show small increases in the coercivity and leakage. As shown in the optical images, the morphology of the device remains almost unchanged, with only some tiny protrusions on the electrode. Besides, there is no significant dark area formed around the electrode edge, suggesting negligible surface oxidation. These results indicate that with the presence of the ionic conductivity only, Cu ions will not be depleted from the sample under AC electric field. Hence, the fatigue process can be effectively suppressed.

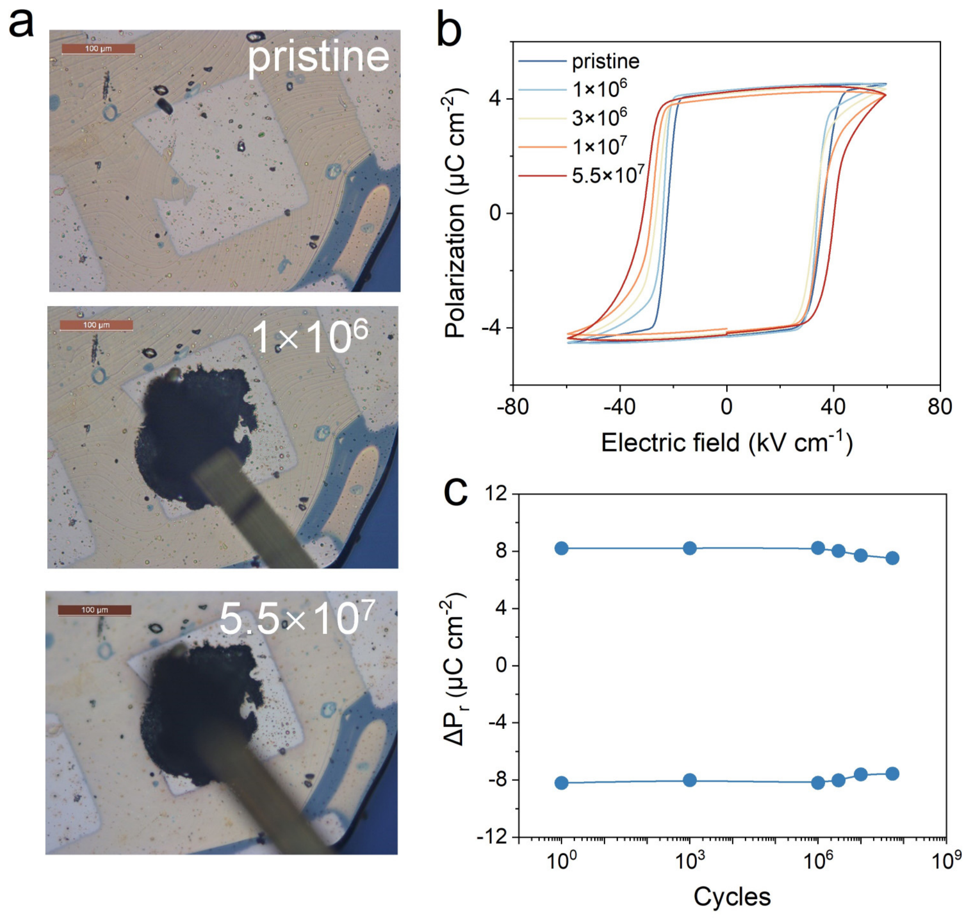

Fig. S19. Fatigue test under vacuum at room temperature. (**a**) Optical images of the capacitor under test at different stages. (**b**) Polarization hysteresis loops at different switching cycles. (**c**) Switchable remanent polarization as a function of electric cycles.

References

1. Brehm, J.A. et al. Tunable quadruple-well ferroelectric van der Waals crystals. *Nat. Mater.* **19**, 43-48 (2020).
2. Kim, S., Seol, D., Lu, X., Alexe, M. & Kim, Y. Electrostatic-free piezoresponse force microscopy. *Sci. Rep.* **7**, 41657 (2017).
3. Gomez, A., Puig, T. & Obradors, X. Diminish electrostatic in piezoresponse force microscopy through longer or ultra-stiff tips. *Appl. Surf. Sci.* **439**, 577-582 (2018).
4. Gannepalli, A., Yablon, D.G., Tsou, A.H. & Proksch, R. Mapping nanoscale elasticity and dissipation using dual frequency contact resonance AFM. *Nanotechnology* **22**, 355705 (2011).
5. Jonscher, A.K. The 'universal' dielectric response. *Nature* **267**, 673 (1977).
