# Supplementary material for: Unconventional polarization fatigue in van der Waals layered ferroelectric ionic conductor CuInP2S6
Source: Nat Commun. 2023 Dec 12;14:8254. doi: 10.1038/s41467-023-44132-y (PMC10716391; doi:10.1038/s41467-023-44132-y)
Supplement: Supplementary file 3 — Description of Additional Supplementary Files [file 41467_2023_44132_MOESM3_ESM.pdf]

Description of additional supplementary files

Supplementary Movie 1 – Time-lapse movie showing the morphological change of CuInP2S6 capacitor under repetitive voltage cycles.

Supplementary Movie 2 – Real time movie showing the process for scratching a bubble protrusion using micromanipulator probe.

Supplementary Movie 3 – Animation of atomic motions for P1 vibrational mode.

Supplementary Movie 4 – Animation of atomic motions for P2 vibrational mode.

Supplementary Movie 5 – Animation of atomic motions for P3 vibrational mode.

Supplementary Movie 6 – Animation of atomic motions for P4 vibrational mode.
